# A Cell Junctional Protein Network Associated with Connexin-26

**DOI:** 10.3390/ijms19092535

**Published:** 2018-08-27

**Authors:** Ana C. Batissoco, Rodrigo Salazar-Silva, Jeanne Oiticica, Ricardo F. Bento, Regina C. Mingroni-Netto, Luciana A. Haddad

**Affiliations:** 1 Human Genome and Stem Cell Research Center, Department of Genetics and Evolutionary Biology, Instituto de Biociências, Universidade de São Paulo, 05508-090 São Paulo, Brazil; rodrigo.salazar.silva@usp.br (R.S.-S.); renetto@ib.usp.br (R.C.M.-N.); haddadL@usp.br (L.A.H.); 2 Laboratório de Otorrinolaringologia/LIM32, Hospital das Clínicas, Faculdade de Medicina, Universidade de São Paulo, 01246-903 São Paulo, Brazil; jeanneoiticica@bioear.com.br (J.O.); rbento@gmail.com (R.F.B.)

**Keywords:** connexin, connexin 26, *GJB2* gene, deafness, protein-protein interaction, TJP1, CGN, FLNB, DAAM1, organ of Corti

## Abstract

*GJB2* mutations are the leading cause of non-syndromic inherited hearing loss. *GJB2* encodes connexin-26 (CX26), which is a connexin (CX) family protein expressed in cochlea, skin, liver, and brain, displaying short cytoplasmic N-termini and C-termini. We searched for CX26 C-terminus binding partners by affinity capture and identified 12 unique proteins associated with cell junctions or cytoskeleton (CGN, DAAM1, FLNB, GAPDH, HOMER2, MAP7, MAPRE2 (EB2), JUP, PTK2B, RAI14, TJP1, and VCL) by using mass spectrometry. We show that, similar to other CX family members, CX26 co-fractionates with TJP1, VCL, and EB2 (EB1 paralogue) as well as the membrane-associated protein ASS1. The adaptor protein CGN (cingulin) co-immuno-precipitates with CX26, ASS1, and TJP1. In addition, CGN co-immunoprecipitation with CX30, CX31, and CX43 indicates that CX association is independent on the CX C-terminus length or sequence. CX26, CGN, FLNB, and DAMM1 were shown to distribute to the organ of Corti and hepatocyte plasma membrane. In the mouse liver, CX26 and TJP1 co-localized at the plasma membrane. In conclusion, CX26 associates with components of other membrane junctions that integrate with the cytoskeleton.

## 1. Introduction

The *GJB2* gene encodes connexin 26 (CX26), which is a protein that plays central roles in hearing, promoting cochlear development, and sustaining auditory function in the mature cochlea [1,2,3,4]. In addition to the cochlea, expression of CX26 is also observed in the skin, the liver, the brain, the mammary gland, the salivary gland, the uterus, testis, the pancreas, lungs, the stomach, the thyroid, and the parathyroid [5]. *GJB2* mutations are the most frequent cause of non-syndromic recessive hearing loss across diverse populations [6,7,8,9]. In addition, some heterozygous *GJB2* mutations behave in a dominant fashion, which leads to non-syndromic autosomal dominant hearing loss or to the keratitis-ichthyosis-deafness syndrome [10].

In vertebrates, connexins (CX) assemble intercellular gap junctions (GJ), which result from the interaction between two distinct hemi-channels from adjacent cells with each composed of six CX units. GJ directly allows the passage of various small (<2 kDa) molecules between two cells such as ions, secondary messengers, nucleotides, amino acids, and short RNAs [11]. GJ are highly organized structures in which CX interact among themselves as well as with a number of other cellular components including cytoskeleton-associated elements and adhesion and signaling molecules [12,13,14]. While, among CX family members, the C-termini are dissimilar and present unique binding partners and signaling, they may share common protein interactors [15,16,17]. The C-terminus from CX26 is strikingly different from that of other CX [18]. Among mouse CX family members, CX26 has the second lowest molecular mass due to shorter segments outside the four transmembrane domains (the extracellular and intracellular loops as well as N-termini and C-termini). Due to its limited length, few binding partners have been identified for CX26 cytosolic segments, e.g., amino-termini and carboxyl-termini and the loop between the second and third transmembrane domains [19,20,21].

The aim of this study was to search for proteins that interact with the cytoplasmic ten-residue carboxyl-terminal tail of CX26. Employing two distinct biochemical approaches, we disclosed a cytoskeleton and membrane junction-associated protein network that co-fractionates with CX26. CX26 interaction with the molecular complex depends on its C-terminus. Additionally, our results revealed that proteins from this macromolecular complex may also associate with CX30, CX31, or CX43, which indicates that assembly of CX in the macromolecular complex is independent of the CX C-terminus length or sequence.

## 2. Results

We employed affinity precipitation assays to search for proteins that interact with the cytoplasmic carboxyl-terminal tail of CX26. To that end, the portion of the *GJB2* mouse gene coding for the 10 most C-terminal amino acids of Cx26 was cloned and expressed in *Escherichia coli* as a peptide in fusion with the glutathione-*S*-transferase (GST) C-terminus (GST–CX26). The purified fusion protein or GST was submitted to affinity capture assays. Mass spectrometry analyses identified 447 proteins from the mouse brain or liver that precipitated in sepharose beads conjugated to glutathione and bound by affinity to the GST–CX26 fusion protein or only GST. After exclusion of potential contaminants, 39 proteins were found to co-fractionate in the GST–CX26 assay but not in the negative control (GST-only assay). The number of peptides identified by mass spectrometry for each of the 39 proteins varied from two to seven and the protein coverage by peptides ranged from 1% to 15%. The number of unique interactor candidates was reduced from 39 to 26 proteins when the following exclusion criteria were applied: redundancy of representation within the GST–CX26 group, discrepancy between the observed and expected molecular weights, and inconsistency in tissue/cell spatial distribution. For instance, biglycan, canstatin, and fibronectin were excluded because, as secreted fibrous proteins, the interaction results would probably be false-positive due to unspecific precipitation or a transient association during synthesis and trafficking in the secretory pathway. As a result, we retrieved a total of 26 candidate proteins to interact with the cytosolic C-terminus of CX26.

**Figure 1:**
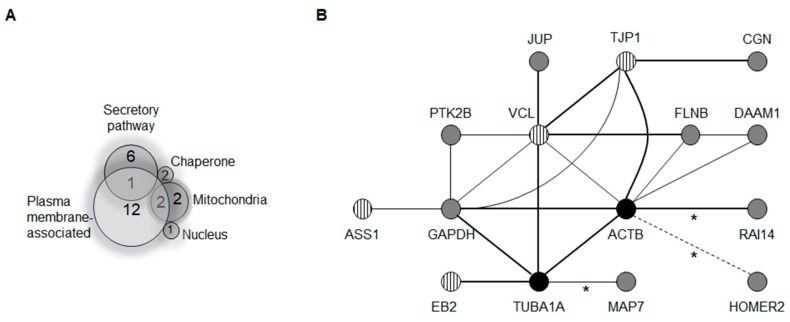
(**A**) Venn diagram representing the distribution of 26 candidate proteins to interact with CX26. The subcellular classification and the number of proteins in each of the five distinct groups are indicated as well as their overlapping sorting; (**B**) The network of protein-protein interaction (PPI) is predicted at http://string-db.org. Acronyms are according to the genes listed on Table 1 in addition to actin B (ACTB), α-tubulin (TUBA1A), and arginine succinate synthase 1 (ASS1). Black circles represent proteins absent from the list of interactors fed by the user to the software input list. Asterisks indicate protein interactions manually added to the PPI network according to the literature (MAP7, RAI14, Homer2). Striped circles denote the four CX26 interactor candidates previously demonstrated to associate with other CX. Thicker lines indicate experimentally demonstrated associations that may be either more reproducible or a direct interaction. A dashed line indicates inference of an intermediate interactor.

Gene ontology and scientific literature searches allowed us to classify the 26 interactor candidates in the following groups: (i) 12 proteins with direct or indirect association with cell junctions and/or the cytoskeleton (Table 1); (ii) seven proteins from the secretory pathway; (iii) four mitochondrial proteins; (iv) two chaperone proteins; and (v) one nucleus-cytoplasm shuttling protein. Two proteins from the mitochondrion group and one protein from the secretory pathway have been detected at the plasma membrane. Therefore, 15 out of 26 proteins may be directly or indirectly associated with the plasma membrane (12 from cell junctions or the cytoskeleton, two from the mitochondrial group, and one from the secretory group). A general subcellular classification of the 26 proteins is illustrated in Figure 1A.

### 2.1. A Membrane-Cytoskeleton Protein Network Associated with CX26

We continued the in silico analyses by verifying the potential ability of the 26 proteins to assemble protein-protein interaction (PPI) networks according to data from literature reports on the interaction and co-expression experiments [22]. Nine out of 26 proteins comprised a single PPI network (ASS1, CGN, DAAM1, FLNB, GAPDH, JUP, PTK2B, VCL, and TJP1). When cytoskeleton proteins α-tubulin and actin were included on the protein list, the PPI network encompassed 10 out of the 26 proteins (EB2 was included). A further individual literature data search allowed for the manual inclusion of three additional proteins in the PPI network (HOMER2, MAP7, and RAI14) (Figure 1B) [23,24,25,26,27]. Consequently, among the 26 proteins, 13 constitute a single PPI network along with α-tubulin and actin (Figure 1B). The PPI network contains all 12 proteins classified as cell junction/cytoskeleton (Table 1, Figure 1B) and one from the mitochondrion group (argininosuccinate synthase 1—ASS1, Figure 1A), which has been reported to interact with the plasma membrane in addition to the mitochondrial outer membrane [28]. In the present study, we report the analysis of the group of 13 proteins present in the PPI network.

### 2.2. Known CX Binding Partners in the CX26 Molecular Complex

Four of the 13 proteins present in the PPI network (Figure 1B, striped circles) have been described to interact with other members from the CX family. They are ASS1, microtubule-associated RP/EB family member 2 (EB2), tight junction protein 1/zonula occludens protein 1 (TJP1), and vinculin (VCL) [29,30,31,32,33,34]. While the former classifies as mitochondria-associated and plasma membrane-associated, the latter three proteins are cell junction or cytoskeleton proteins. As CX interaction with TJP1 appears to be direct for the majority of family members studied [29,30,31,35,36,37,38,39,40], we adopted the yeast two-hybrid split-ubiquitin system to search for direct, pairwise interaction between full-length CX26 individually with TJP1, ASS1, EB2, and VCL.

**Figure 2:**
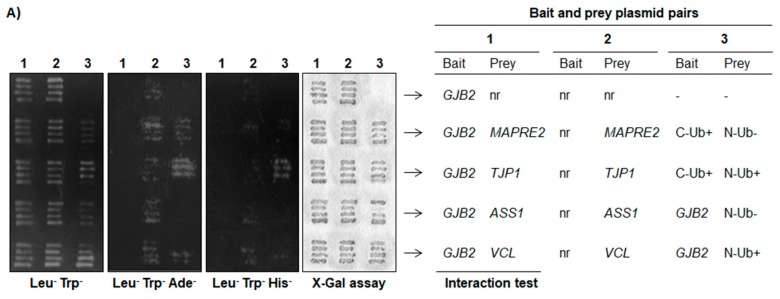

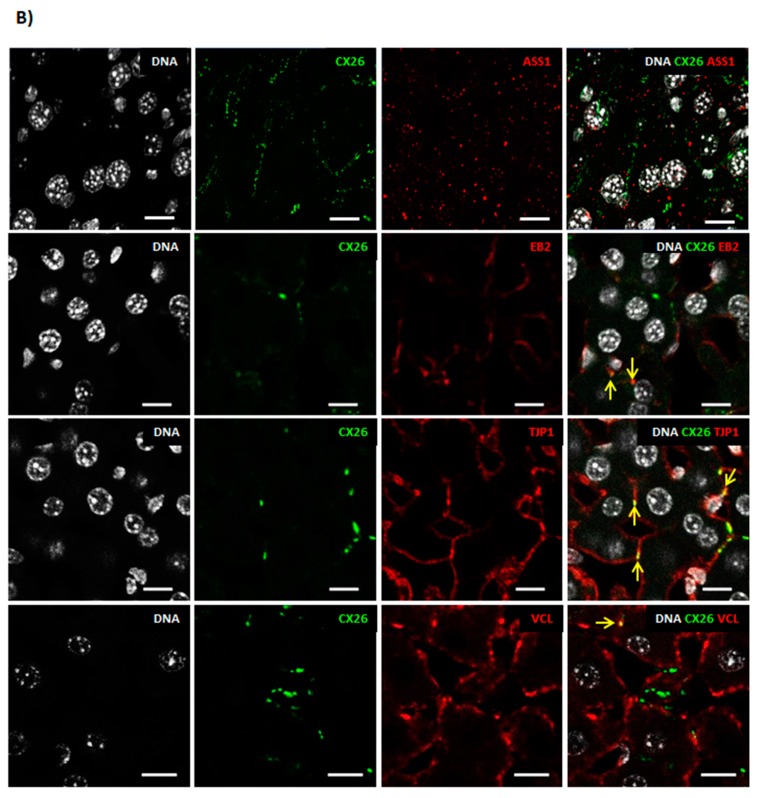
(**A**) Results of the yeast two-hybrid split ubiquitin assay has, in column 1, interaction tests with *GJB2* full-length sequence as bait and the preys non-recombinant vector (nr, control), EB2, TJP1, ASS1, or VCL. Column 2 has the non-recombinant (nr) vector as bait. Therefore, it leads to testing for leaky activation of the reporter gene by each prey fusion protein. Column 3 presents different controls: untransformed, positive, and negative controls for each vector (coding for either N- (N-Ub) or C-terminal (C-Ub) ubiquitin moieties in fusion with known strong (+) or negative (−) interactors. Different media respectively select for the presence of both vectors (SD, Leu^−^, Trp^−^), activation of the reporter gene *ADE2* (SD, Leu^−^, Trp^−^, Ade^−^), *HIS3* (SD, Leu^−^, Trp^−^, His^−^), or β-galactosidase (X-Gal test); (**B**) Indirect immunofluorescence of adult (P60) mouse liver cryosections with anti-CX26 antibody (green) and ASS1, VCL, EB2, and TJP1 (all in red). DNA is in a pseudo white color, according to DAPI staining. The analysis was performed at confocal microscopy with *z*-sections of 0.5 µm (LSM880, Carl Zeiss, Oberkochen, Germany). Arrows: merged signals. Scale bar: 10 µm.

In the yeast split-ubiquitin system, the interaction is expected to take place at the membrane and cleavage of the fusion protein by a ubiquitin-specific processing protease and then releases the transcription factor lexA-VP16. The reporter genes *lacZ*, *HIS3*, and *ADE2* were employed in this study as they are responsive to lexA-VP16 binding after its nuclear translocation. As presented in Figure 2A, no specific activation of the reporter genes was observed for any test bait-prey pair (CX26–TJP1, CX26–VCL, CX26–EB2, or CX26–ASS1). Leaky activation was observed for the *lacZ* gene expression for all pairs and the *ADE2* gene was activated by the preys themselves. No test pair allowed for yeast growth in minimal medium without histidine when compared to the positive control (Figure 2A). Therefore, we concluded that, under these conditions, we did not obtain data indicating direct interaction between full-length CX26 and TJP1, VCL, EB2, or ASS1.

Antibodies that recognize each of the four CX interactors including TJP1, VCL, EB2, and ASS1 were employed in immunofluorescence assays of adult mouse liver with double staining for CX26 in all four experiments. CX26, TJP1, VCL, and EB2 all presented staining patterns consistent with plasma membrane distribution in hepatocytes (Figure 2B). TJP1 and CX26 co-localized in different points at the plasma membrane (Figure 2B, arrows). On the other hand, although in double staining assays, there were sporadic merged signals between CX26 and either EB2 or VCL (Figure 2B, arrows), we considered that VCL and EB2 do not co-localize with CX26 in the mouse liver under our assay conditions. ASS1 disclosed a cytoplasmic staining pattern and no co-localization with CX26 under the conditions employed (Figure 2B). However, in a control experiment, ASS1 did not co-localize with the mitochondrial cell marker mtHSP70 (data not shown), which suggests that the anti-ASS1 antibody employed should be unspecific in our immunofluorescence assays of mouse liver sections.

### 2.3. Expanding the Protein Interaction Network with Cx26

**Figure 3:**
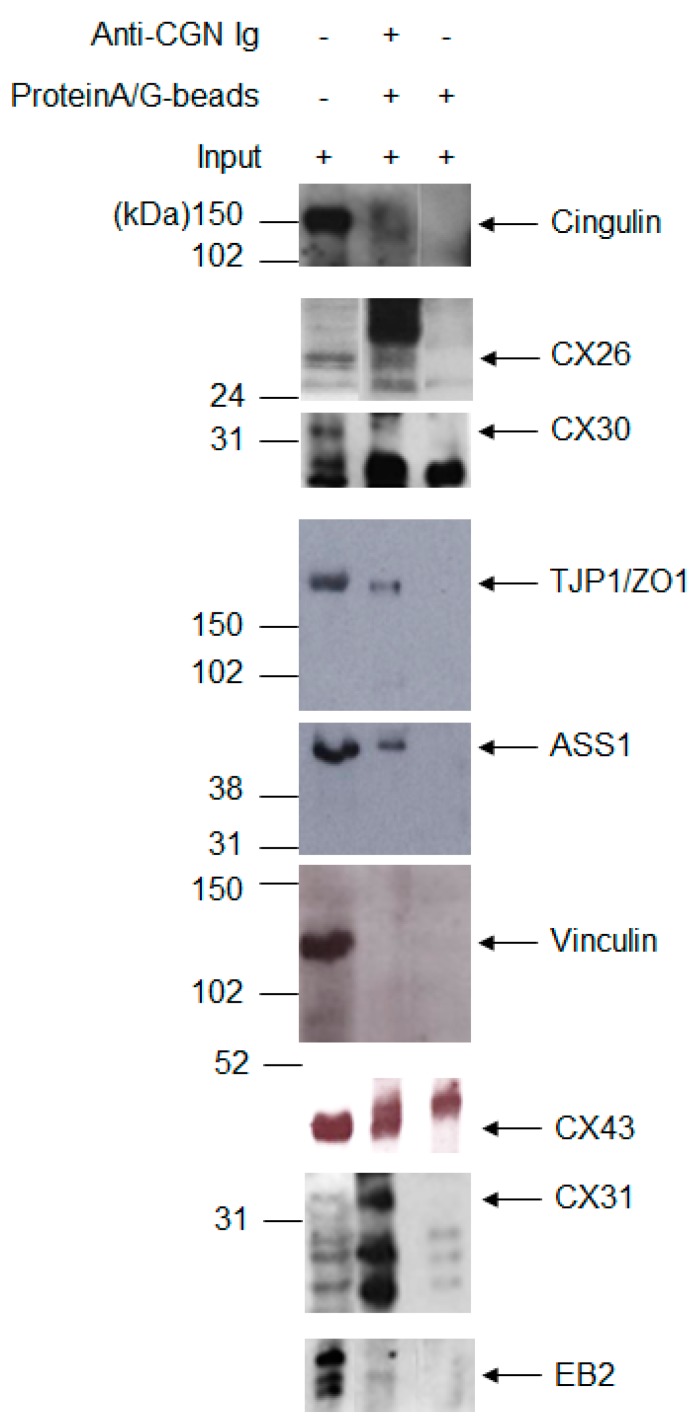
Immunoprecipitation of cingulin from adult mouse liver and its co-immunoprecipitation with CX26, CX30, CX31, CX43, EB2, TJP1, and ASS1, which is indicated by the respective arrows. No co-immunoprecipitation is observed with VCL. The protein molecular mass is indicated in kiloDaltons (kDa).

Since some CX26 interactor candidates retrieved in our affinity capture assay are common to other CX and do not appear to be in direct association with CX26, we looked for adaptor proteins in the PPI network that could play roles in bringing TJP1, VCL, ASS1, or EB2 to proximity to CX26. Among the 13 proteins from the PPI network, there are four adaptor/scaffolding proteins: cingulin (CGN), filaminB (FLNB), dishevelled-associated activator of morphogenesis 1 (DAAM1), and homer scaffolding protein 2 (HOMER2). As the N-terminal globular domain of the adaptor protein CGN associates with TJP1 PDZ domain [41], we performed immunoprecipitation experiments with anti-CGN antibodies in RIPA or EGTA buffer lysates of neonate mouse liver tissue and confirmed that CGN, as expected, co-immunoprecipitates with TJP1. We confirmed co-immunoprecipitation of CGN and CX26 in both buffer compositions employed, RIPA buffer (Figure 3), and EGTA buffer (data not shown). ASS1 and EB2 were disclosed to co-immunoprecipitate with CGN. Data presented in Figure 3 are clear evidence for CGN co-immunoprecipitation with TJP1 and ASS1. The faint band observed for EB2 in a CGN immunoprecipitation lane led us to consider it may be due to weak and indirect association with proteins from any CGN protein complex. We did not observe VCL in CGN immunoprecipitates in either buffer condition (Figure 3 and data not shown) even though it may still bind CX26 through an additional intermediate interactor. Although CX43-specific blot band migrates very close to an unspecific band observed in the negative control, its co-immunoprecipitation with CGN is demonstrated (Figure 3). Lastly, we show that CGN co-immunoprecipitates with CX30 and CX31 (Figure 3).

**Figure 4:**
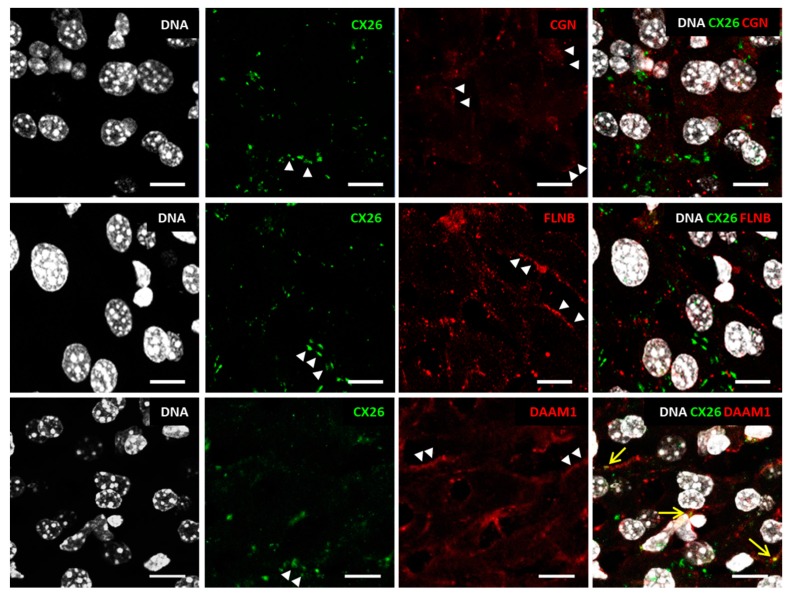
Indirect immunofluorescence of adult (P60) mouse liver cryosections with anti-Cx26 antibody (green) and CGN, FLNB, and DAAM1 antibodies (all in red). DNA is in a white pseudo color, according to DAPI staining. The analysis was performed at confocal microscopy with *z*-sections of 0.5 µm (LSM880, Carl Zeiss, Oberkochen, Germany). Each image consists of the maximum intensity projection of all z-sections obtained. Arrowheads: signal at the plasma membrane. Arrows: merged signals. Scale bar: 10 µm.

**Figure 5:**
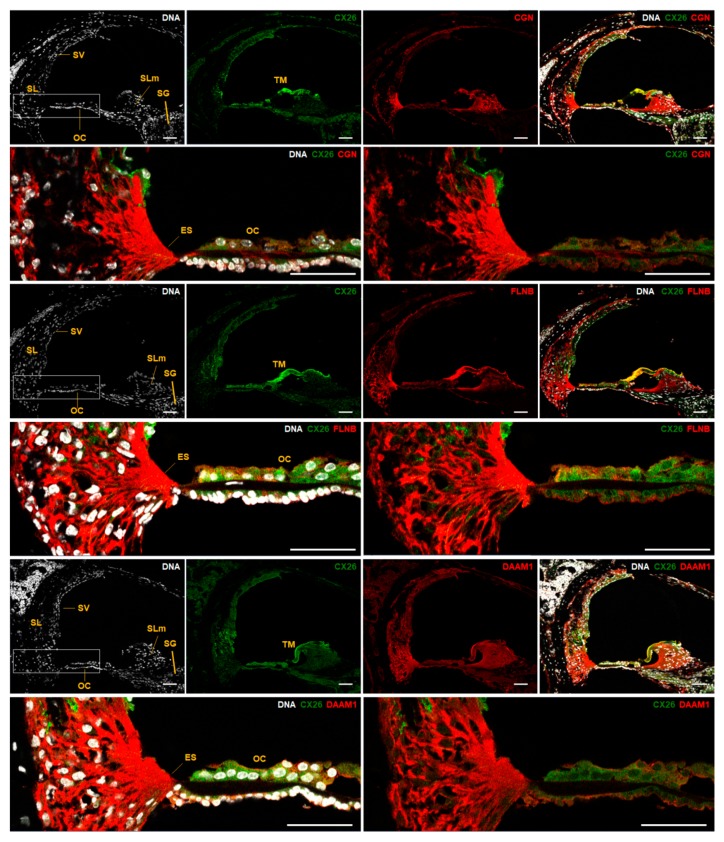
Indirect immunofluorescence of P14 mouse cochlea cryosections and zooming-in at the organ of Corti (OC) indicated by a rectangle. The sections highlight the spiral ligament (SL), *stria vascularis* (SV), spiral limbus (SLm), spiral ganglion (SG), external sulcus cells (ES), OC, and tectorial membrane (TM). Labeling by anti-Cx26 antibody (green) and antibodies for CGN, FLNB, and DAAM1 (all in red) are presented. DNA is in a white pseudo color, according to DAPI staining. The analysis was performed at confocal microscopy with z-sections of 1 µm (LSM880, Carl Zeiss, Oberkochen, Germany). Each image consists of maximum intensity projection of all z-sections obtained. Scale bar: 50 µm.

The adult mouse liver was co-labeled for Cx26 and three adaptor proteins known as CGN, FLNB, or DAAM1. The four proteins were detected in hepatocytes and in patterns consistent with plasma membrane localization (Figure 4, arrowheads) as well as cytoplasm organelles (Figure 4). Few overlapping signals were observed in hepatocytes for CX26 and DAAM1 at the plasma membrane (Figure 4, arrows). The paucity of these observations led us to exclude co-localization of those proteins. CX26 co-localization with CGN or FLNB was not observed under those conditions. Moreover, we show that all three adaptor proteins including CGN, FLNB, and DAAM1 are expressed in key regions of P14 mouse cochlea such as the organ of Corti (OC), *stria vascularis*, spiral ligament, spiral limbus, and external sulcus cells (Figure 5). DAAM1 is also present in spiral ganglion cells (Figure 5). Likewise, in *stria vascularis* DAAM1, staining appeared more evident than CGN and FLNB staining. On P14, CX26 distributed to the OC, *stria vascularis*, spiral ligament, spiral limbus, and the spiral ganglion. The tectorial membrane is acellular and known to yield unspecific staining due to spontaneous fluorescence. CX26 and all three adaptor proteins seem to be co-expressed in the OC even though OC cell identity could not be precisely defined since distinct cell type markers have not been employed.

## 3. Discussion

CX26 assembly as heteromeric hemi-channels and heterotypical gap junctions has been demonstrated in particular through its association with CX30 [42]. CX26 association with other CX has also been disclosed by global interactome analyses [33]. CX26 physical interaction with paralogues is, therefore, a common feature since it is for other family members [43]. Few additional binding partners have been reported for CX26. The trans-Golgi network protein consortin interacts with CX26 in the secretory pathway [21]. At the plasma membrane, CX26 binding to caveolin-1 is necessary for its localization in caveolae from lipid rafts [44]. Lastly, CX26 association with dynamin-2 has been implicated in its turnover by endocytosis [45]. The finding of CX26 interaction with the SCF E3 ubiquitin ligase component known as the F-box protein OCP1 has also contributed to clarify its turnover mechanism [46]. As seen, few proteins are known as binding partners of CX26. Therefore, we employed the CX26 C-terminus as bait and sought for interacting proteins from the adult mouse brain or liver.

In this paper, we presented 13 proteins that have been identified by mass spectrometry analysis of the CX26 C-terminus affinity precipitation assays with 12 of them having been classified as cell junction and cytoskeleton-associated proteins (Table 1). Four proteins have previously been identified as other CX interactors (ASS1, EB2, TJP1, VCL, Figure 1B). Three proteins from this subgroup are part of cell junctions and the cytoskeleton (EB2, TJP1, and VCL). TJP1 directly interacts with the C-termini of CX30, CX31.9, CX32, CX35, CX36, CX43, CX45, CX46, CX47, and CX50 [29,30,31,35,36,37,38,39,40] as well as with VCL (Figure 1B) [34] and is important to stabilize CX43 gap junctions [34]. Moreover, EB1, which is a paralogue of EB2, has been shown to be necessary for targeting CX26 and CX43 to the plasma membrane and co-immunoprecipitates with CX43 [32].

TJP1 is a large protein with three tandem N-terminal PDZ domains, which mediate its interaction with CX. TJP1 binding to CX C-terminus is an important regulatory step in a gap junction assembly, internalization, and degradation [47]. Apparently, TJP1 binding needs a CX C-terminus to be anchored at the membrane or protein complex. For affinity capture, we employed CX26 C-terminus in fusion with the GST C-terminus. This configuration may have contributed to in vitro binding of TJP1 to the GST–CX26 C-terminus. However, contrary to other connexins such as CX43, CX26 does not have a PDZ-binding motif in its C-terminus (data not shown). In fact, PDZ-binding motifs need to be internal at the C-terminus to properly mediate protein interaction [48] and the CX26 C-terminus is only 11-amino acids long. Therefore, it was not surprising that the yeast two-hybrid system did not reveal a direct interaction between TJP1 and CX26 (Figure 2A). We then looked at indirect interactions between these proteins and employed CGN in immunoprecipitation assays. There is a direct association between the N-terminal globular domain of the adaptor protein CGN and the TJP1 PDZ domain [41] and we demonstrated that CX26 co-immunoprecipitates with CGN (Figure 3). No extensive co-localization was observed between CGN and CX26 in the liver (Figure 4) or cochlea (Figure 5). Therefore, a direct interaction between these proteins is not suggested by our data.

Due to the limited length of the CX26 C-terminus, other scenarios could be considered to speculate how CX26 interactions described here would take place. Each hemi-channel displays six CX N-termini, six intracellular loops, and six C-termini at the cytosolic face of the plasma membrane. Hence, it is plausible that the CX26 C-termini combine in specific configurations that allow for protein interactions, which individually would not be possible. Those segments could be stabilized through an interaction with other membrane proteins or the lipid content from the plasma membrane in a stoichiometric ratio likely different from one-to-one.

**Figure 6:**
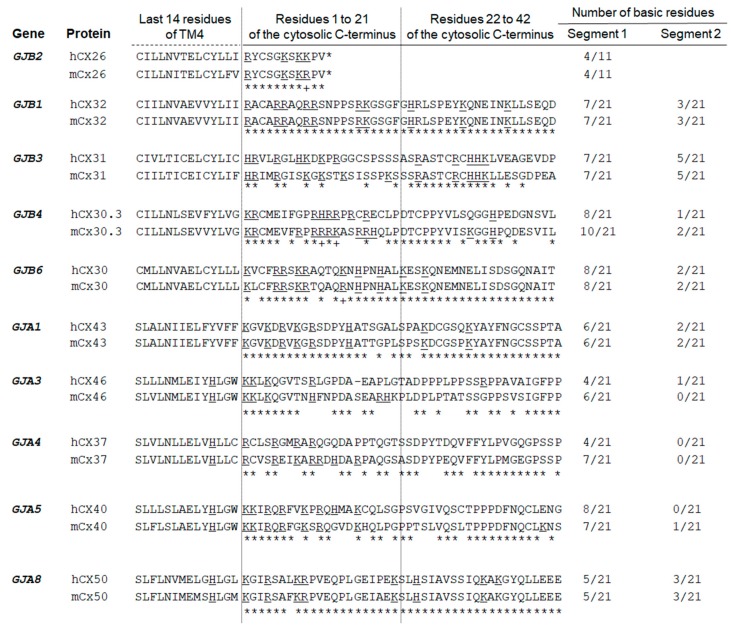
Pairwise alignments of human (h) and mouse (m) CX orthologues as indicated by comprehending part of the last transmembrane domain (TM4) and 42 amino acids that follow that domain. Basic amino acids are underlined and their counting indicated on the two right columns, respectively, for the first and second 21-residue initial segments of C-termini. (*) identical residues; (+) amino acids with lateral chains from the same biochemical group.

Multiple sequence alignments among CX C-termini display limited similarity between the short cytosolic C-termini of CX26 and other B-group CX paralogues (data not shown). On the other hand, manual alignments between human and mouse CX26 11-residue C-terminus and up to 42 residues of the C-termini from the A-group and the B-group CX disclosed enrichment (30% to 50%) in basic amino acids in the first 21 cytosolic amino acids of all CX analyzed (Figure 6). Functional roles in the gap junction sensitivity have been proposed for the CX32 C-terminus basic residues [49]. Although basic residues are commonly observed in cytosolic protein segments contiguous with transmembrane domains and by balancing the net negative charge of the lipid bilayer, they may also mediate common interactions with scaffolding proteins [50]. Therefore, an additional scenario would be that the basic amino acids of at least two CX26 C-termini help to stabilize molecular interactions in the PPI network in close adjacency to the membrane. This network could be assembled on the cytoplasmic face of membranes at any point along the secretory pathway.

Our results do not indicate a specific interactor that could be a direct binding partner for CX26. Hence, the macromolecular assembly hypothesis is corroborated and it contributes to a protein platform at the cytosolic face of CX26 hemi-channels associated with the membrane and other junction proteins. While interaction between tight junctions and CX has previously been observed solely for CX32 [51], we could detect co-localization of CX26 and TJP1 at the plasma membrane of hepatocytes from the mouse liver (Figure 2B). It is also a possibility that CX26 hemi-channels would associate in vivo with tight junction proteins if composed of heteromeric assemblies. Alternatively, the tight junction proteins could associate with CX26 C-terminus during trafficking at the Golgi cytoplasmic face.

EB2 and VCL disclosed no convincing co-localization with CX26 in the mouse liver (Figure 2B). Specifically, microtubule plus end-binding proteins, EB1 and EB3, have been implicated in microtubule dynamics promoting microtubule growth and inhibiting its catastrophe [52]. In microtubule-assisted disassembly of focal adhesions, microtubule growth is believed to take place on underlying actin microfilaments and associated proteins. It has been demonstrated that EB2 knocking-down decreases cell motility and causes aberrant focal adhesion dynamics. EB2 has been shown to be essential for focal adhesion disassembly as a direct microtubule interactor and through its interaction with MAP4K4 (mitogen-activated protein kinase 4) [53]. Moreover, EB1 plays roles in CX43 trafficking to regions of the plasma membrane where adherens junctions had already been formed [32,54,55].

VCL is a membrane-cytoskeletal protein in focal adhesion plaques involved in the linkage of integrin adhesion molecules to the actin cytoskeleton. It is a cytoskeletal protein associated with cell-cell and cell-matrix junctions where it is thought to function as one of several interacting proteins involved in anchoring F-actin to the membrane [56]. VCL binding to CX43 has already been demonstrated by in vivo and in vitro studies including co-immunoprecipitation and co-localization [57]. Therefore, focal adhesions are cytoskeleton-membrane association sites where CX26 interaction with VCL and EB2 could be investigated.

ASS1 is the fourth protein from the CX26 PPI network that has previously been identified as a CX interactor since it has been detected in the CX32 interactome [33]. Although ASS1 is not part of the cell junction or the cytoskeleton, it has been analyzed in this report since it distributes to the plasma membrane of endothelial cells. More specifically, associated with endothelial nitric oxide synthase in caveolae from lipid rafts [28], where CX26 has also been identified [44]. However, most commonly, ASS1 is described in the vicinity of the mitochondria outer membrane [58]. ASS1 and other enzymes from the urea cycle are believed to form a macromolecular complex that facilitates and concentrates arginine metabolism components near mitochondria. Since ASS1 gene expression and ASS1 protein localization have been demonstrated to be regulated by hormones and amino acids [58], it is logical to assume that, when driven to caveolae, ASS1 association to cell membrane junction proteins such as CX and paralogues would be more pronounced. On the other hand, a few reports have implicated CX in mitochondrial functions. CX43 has been shown to localize in the mitochondria inner membrane [59] where it is assembled as a hemi-channel and functions in homeostasis and cell death [33,60]. Therefore, on the one hand, plasma membrane caveolae are a likely address for interaction between CX and ASS1. On the other hand, although CX43 and ASS1 have been reported in different mitochondria compartments including mitochondrial inner and outer membranes, indirect interaction could take place between CX and ASS1 during the transport to mitochondria. This is probably a considerable alternative since we did not observe co-localization of CX26 and ASS1 at the plasma membrane (Figure 2B).

We showed that CGN, DAAM1, and FLNB distribute to the organ of Corti (Figure 5). Moreover, all 13 proteins from the CX26 PPI network have been reported to be expressed in the inner ear, according to databases [61,62,63,64,65]. Among the 13 genes that encode CX26 interactors, the HOMER2 gene has been related to autosomal dominant hearing loss in humans with the description of a missense mutation [66]. In addition, tricellulin, which is a protein encoded by the *TRIC* gene, presents in its C-terminal region a domain for binding to occludin, which is known as a TJP1 direct binding partner [67]. Protein-truncating mutations in the *TRIC* gene led to the loss of the occludin-binding domain and autosomal recessive hearing loss in humans [68]. In the inner ear, tricellulin is in cell junctions of supporting and ciliated cells. These data corroborate our results on TJP1 as part of the CX26 interactome. Lastly, the localization of CX26 and adaptor proteins belonging to its cell junctional network in the cochlea confirms their potential for physiological roles in hearing. Their corresponding genes are unveiled as good candidates to be explored in hearing loss studies. Among other functions, they may participate in the mechano-electrical transduction of sound vibrations in the organ of Corti [69] or in the maintenance of cochlear ion homeostasis regulated through *stria vascularis* [70].

## 4. Materials and Methods

### 4.1. Animals and Tissue

The experimental protocol was previously approved by the Internal Review Board on Ethics in Animal Research from the Institute of Biosciences of the University of São Paulo (CEP 062/2007, 3 April 2007). All experiments were conducted in accordance with the guidelines for the care and use of laboratory animals established by the American National Research Council. Postnatal day 3 (P3) Balbc mice (*Mus musculus*), postnatal day 14 (P14), and 60 (P60) CBL57/6 mice (*Mus musculus*) were obtained from specialized breeders from the University of São Paulo (São Paulo, Brazil). Animals presenting acute or chronic ear infection or congenital malformations were excluded from the study.

The P60 CBL57/6 mouse brain and liver were obtained for affinity precipitation assays. P3 mouse liver was employed in immunoprecipitation assays. To obtain the biological material of interest, the animals were submitted to profound anesthesia with ketamine and xylazine (0.17 and 0.03 mg/g of body mass, respectively) followed by decapitation. The heads and abdomen were bathed in 70% ethanol, which was followed by the removal of the liver and sagittal incision of the skull to remove all brain tissue. Tissues were snap-frozen in liquid nitrogen and later stored at −80 °C. P14 CBL57/6 mice provided cochlea for immunofluorescence assays. The animals were sacrificed as described above. The heads were bathed in 70% ethanol, temporal bones were removed, labyrinth dissected, and cochleae was harvested with micro tweezers (Dumont #5 and #54, Electron Microscopy Sciences, Hatfield, PA, USA), under a trinocular stereomicroscope (Discovery V12, Carl Zeiss, Oberkochen, Germany) and incubated in 4% paraformaldehyde. For immunofluorescence assays, P60 CBL57/6 mice were submitted to profound anesthesia with ketamine and xylazine (0.17 and 0.03 mg/g of body mass, respectively), which was followed by trans-cardiac perfusion with 4% paraformaldehyde in phosphate-buffered saline solution (PBS; 137 mM NaCl, 2.7 mM KCl, 10 mM Na_2_HPO_4_, and 1.76 mM KH_2_PO_4_, pH 7.4) at 4 °C.

### 4.2. DNA Clones

To obtain a recombinant pGEX-4T-1 clone containing the DNA coding sequence for glutathione-*S*-transferase (GST) in fusion with that encoding CX26 C-terminus, genomic DNA from mouse liver was used as a template for a polymerase chain reaction (PCR) amplification of human *GJB2* gene DNA from base chr13:2018852 to 20188933 (GRCh37/hg19) with the following pair of primers: (1S) 5′ CGA GGC CCG GGT TAT TGC TCA GGA AAG TCC A 3′ and (1AS) 5′ CGA GGG CGG CCG CTG GGT TCC TCT CTC CTG TC 3′, with the 5′-end having restriction sites respectively for *Sma*I and *Not*I (underlined). The undigested, purified PCR product was cloned in a pCR2.1-TOPO vector (Invitrogen, Carlsbad, CA, USA) in DH5α *E. coli*. Plasmid DNA from one recombinant clone with its insert sequence confirmed by Sanger sequencing was digested with *Sma*I and *Not*I (Invitrogen). A released insert was subcloned in the pGEX-4T-1 vector (GE Healthcare, Little Chalfont, UK) and the final recombinant clone was named PGEX-GST–CX26. For the yeast two-hybrid interaction test, the bait construct was obtained after a cloning mouse *GJB2* full-length coding sequence (present in a single exon) into the vector pBT3-N (MoBiTec, Göttingen, Germany) while prey constructs (full-length coding sequence for mouse VCL, TJP1, ASS1, or MAPRE2) were sub-cloned into the vector pPR3-N (MoBiTec).

### 4.3. Antibodies

The antibodies from Santa Cruz Biotechnology (Santa Cruz, CA, USA) used to detect the following proteins were: CX26 (*GJB2*, goat polyclonal antibody N-19, and rabbit polyclonal antibody O-24), CX30 (GJB6, rabbit polyclonal antibody C20), CX31 (GJB1, rabbit polyclonal antibody H-43), CX43 (GJA1, mouse monoclonal antibody F-7), ASS1 (rabbit monoclonal antibody H-231), CGN (cingulin, mouse monoclonal antibody G-6), DAAM1 (disheveled-associated activator of morphogenesis 1, mouse monoclonal antibody WW-3), FLNB (filamin B, mouse monoclonal antibody F-8), and TJP1 (zonula occludens 1 protein, ZO-1, rat polyclonal antibody R40.76). Antibodies from Abcam (Cambridge, MA, USA) were as follows: MAPRE2 (EB2, rat polyclonal antibody K52), VCL (vinculin, mouse monoclonal antibody SPM227), and TJP1 (rabbit polyclonal antibody ab59720). An additional anti-CX26 (Zymed mouse monoclonal antibody, Thermo Fisher Scientific, Waltham, MA, USA) was employed in immunofluorescence assays. Western blot secondary antibodies were conjugated to horseradish peroxidase (GE Healthcare, Wauwatosa, WI, USA). Immunofluorescence secondary antibodies were conjugated to Alexa488 or Alexa594 (Jackson ImmunoResearch Laboratories, West Grove, PA, USA).

### 4.4. Bacteria Expression of Fusion Protein

For fusion protein expression, pGEX-GST–CX26 or pGEX-GST plasmid DNA was used to transform BL21 *E. coli*. Recombinant clones were grown at 37 °C in liquid LB medium supplemented with 50 µg/mL ampicillin (TEUTO, Anapólis, Brazil) and 0.5 mM IPTG (Invitrogen) until optic density (600 nm) reached values between 0.4 and 0.6. For soluble protein isolation, a bacteria pellet was suspended in PBS with a protease inhibitor (Pefabloc, Roche Applied Science, Indianapolis, IN, USA), 10 mg/mL lysozyme (Sigma-Aldrich, St Louis, MO, USA), and incubated on ice for 15 min. After two quick cycles of freezing and thawing, lysates were centrifuged at 10,000× *g*, for 15 min at 4 °C. One-hundred µL of the supernatant were mixed with 40 µL of GST-Bind™ resin (glutathione (GSH)-sepharose, Novagen, Darmstadt, Germany) under agitation at 4 °C for 30 min. After washing the pellet, samples of sepharose beads containing glutathione-bound proteins were boiled and submitted to SDS-PAGE for protein quantification in comparison to bovine serum albumin (BSA) standards. Bacterium soluble protein fractions containing 600 pmols of GST–CX26 or GST were aliquoted and stored at −80 °C.

### 4.5. Affinity Capture Assay

Three different lysis buffers named EDTA, EGTA, or PHEM were employed for obtaining mouse tissue lysates. For each one, 10 to 20 mg of mouse liver or brain were homogenized in 1 mL of the lysis buffer using a Douncer homogenizer (40 slow strokes). The basic composition of EDTA and EGTA buffers was the same: 50 mM Tris-HCl pH 7.4, 150 mM NaCl, 0.75% triton X-100, 2 mM Na_3_VO_4_, 10 mM NaF, 1× protease inhibitor (cOmplete, EDTA-free, Sigma-Aldrich, St Louis, MO, USA). EDTA and EGTA buffers differed on a divalent cation composition with the former having 1 mM EDTA and the latter 10 mM EGTA and 2 mM MgCl_2_. PHEM buffer was composed of 60 mM piperazine-*N*,*N*′-bis [2-ethane-sulfonic acid] (PIPES) pH 6.9, 25 mM *N*-2-hydroxyethylpiperazine-*N*′-2-ethane-sulfonic acid (HEPES), 2 mM MgCl_2_, 10 mM EGTA, 0.75% triton X-100, 5 µM phallacidin (Sigma-Aldrich, St Louis, MO, USA), 2 mM Na_3_VO_4_, 10 mM NaF, 1× protease inhibitor (complete, EDTA-free, Sigma-Aldrich). After tissue homogenization in EDTA or EGTA buffers, the suspension was incubated on ice for 30 min and then centrifuged for 30 min at a temperature of 4 °C at 14,000× *g*. The supernatant was transferred to a new tube, the protein was quantified, and the lysate was aliquoted and stored at −80 °C. After Douncer homogenization of tissue in PHEM, the suspension was incubated for 2 min at 37 °C and centrifuged for 5 min at a temperature of 4 °C at 14,000× *g*. The pellet was re-suspended in PHEM buffer and, after a novel centrifugation, both supernatants containing soluble proteins were pooled.

For affinity precipitation, 600 pmoles of GST–CX26 or GST were bound to 200 μL of 50% GST™Bind Resin sepharose beads (Novagen) and incubated at RT for 30 min under agitation. After three washes with PBS and protease inhibitor (Pefabloc, Roche Applied Science), lysates were added to the beads and the samples were maintained under rocking at 4 °C for 16 h. The samples were washed in a lysis buffer without triton-X-100, centrifuged and submitted to SDS-PAGE, and followed by Coomassie blue staining, according to standard procedures.

### 4.6. Mass Spectrometry Analyses

Gel bands identified after Coomassie blue staining were compared between the two lanes of SDS-polyacrylamide in which precipitates from GST–CX26 or GST-only had been electrophoresed side by side. Lanes with apparently similar total protein loading had specific gel bands visually compared. Bands with discrepant intensities between the two lanes were excised from the whole gel, which led to a total of 11 gel band pairs. The in-gel digest and mass spectrometry experiments were performed by the Proteomics platform of the Research Center at the Quebec University Hospital Center (CHUQ, Laval University, QC, Canada). Protein from the excised gel bands were digested with trypsin on a MassPrep liquid handling robot (Waters, Milford, CT, USA), according to the manufacturer’s specifications and to the protocol of Shevchenko et al. [71] with the modifications suggested by Havlis et al. [72]. Proteins were reduced with 10 mM DTT and alkylated with 55 mM iodoacetamide. Trypsin digestion was performed using 126 nM of modified porcine trypsin (Sequencing grade, Promega, Madison, WI, USA) at 58 °C for 1 h. Digestion products were extracted using 1% formic acid, 2% acetonitrile followed by 1% formic acid, and 50% acetonitrile. The recovered extracts were pooled, vacuum centrifuge-dried, and then suspended into 7 µL of 0.1% formic acid and 2 µL were analyzed by mass spectrometry.

The resulting peptides were separated by online reversed-phase (RP) nanoscale capillary liquid chromatography (nanoLC) and analyzed by electrospray mass spectrometry (ES MS/MS). The experiments were performed with a Thermo Surveyor MS pump connected to a LTQ linear ion trap mass spectrometer (Thermo Fisher, San Jose, CA, USA) equipped with a nano-electrospray ion source (Thermo Fisher). Peptide separation took place on a self-packed PicoFrit column (New Objective, Woburn, MA, USA) packed with a C18 Jupiter HPLC column (5-µm particle size, 300-Å pore size; Phenomenex, Torrance, CA, USA). Peptides were eluted with a linear gradient of 2–50% acetonitrile, 0.1% formic acid in 30 min at 200 nL/min (obtained by flow-splitting). Mass spectra were acquired using a data-dependent acquisition mode using Xcalibur software (Version 2.0, Thermo Fisher Scientific). Each full scan mass spectrum (400 to 2000 *m*/*z*) was followed by collision-induced dissociation of the seven most intense ions. The dynamic exclusion (30-s duration) function was enabled and the relative collisional fragmentation energy was set to 35%.

All MS/MS samples were analyzed using Mascot (Version 2.3.0; Matrix Science, London, UK). The Mascot was set up to search the Uniref100 mouse database (release of June 2010; 80,419 entries) when assuming the digestion by trypsin. Mascot was searched with a fragment ion mass tolerance of 0.50 Da and a parent ion tolerance of 2.0 Da. Iodoacetamide derivative of cysteine was specified as a fixed modification and oxidation of methionine was specified as a variable modification. Two missed cleavages were allowed.

Scaffold (Version 3.6.2; Proteome Software Inc., Portland, OR, USA) was used to validate MS/MS based peptide and protein identifications. Protein identifications were accepted if they could be established at greater than 95% probability and contained at least two identified peptides, which is specified by the Peptide Prophet algorithm [73]. Proteins that contained similar peptides and could not be differentiated based on the MS/MS analysis alone were grouped to satisfy the principles of parsimony.

### 4.7. NCBI Protein Reference Sequence Accession Numbers

Initial protein identification was completed according to Uniprot (http://www.uniprot.org/) accession numbers. Corresponding NCBI mouse protein RefSeq accession numbers were as follows: ASS1 (argininosuccinate synthase, NP_031520.1), CGN (cingulin, NP_001032800.2), DAAM1 (disheveled-associated activator of morphogenesis, NP_766052.2), FLNB (filamin-B, NP_001074896.1), GAPDH (glyceraldehyde-3-phosphate dehydrogenase, NP_001276675.1), Homer2 (homer protein homolog 2 isoform 1: NP_036113.1), JUP (junction plakoglobin, NP_034723.1), MAP7 (ensconsin, NP_032661.2), MAPRE2 (microtubule-associated protein RP/EB family member 2, NP_694698.3), PTK2B (protein tyrosine kinase 2β, NP_001155838), RAI14 (ankycorbin, NP_001159880.1), TJP1 (tight junction protein, NP_033412.2), and VCL (vinculin, NP_033528.3). RefSeq accession numbers for human and mouse CX protein paralogues employed in multiple sequence alignments were: connexin 26 (hCX26 NP_003995.2, mCX26 NP_032151.1), connexin 30 (hCX30 NP_001103689.1, mCX30 NP_001258592.1), connexin 30.3 (hCX30.3 NP_694944.1, mCX30.3 NP_032153.1), connexin 31 CX31 (hCX31 NP_001005752.1, mCX31 NP_001153484.1), connexin 32 (hCX32 NP_000157.1, mCX32 NP_032150.2), connexin 37 (hCX37 NP_002051.2, mCX37 NP_032146.1), connexin 40 (hCX40 NP_859054.1, mCX40 NP_001258557.1), connexin 43 (hCX43 NP_000156.1, mCX43NP_034418.1), connexin 46 (hCX46 NP_068773.2, mCX46, NP_001258552.1), and connexin 50 (hCX50 NP_005258.2, mCX50 NP_032149.1).

### 4.8. In Silico Analyses

Computational analyses of nucleic acids and proteins were performed at the following web sites, according to their recommendations: EMBL-EBI [74], ExPASy Proteomics server [75], Hereditary Hearing Loss Homepage [76], Kyte Doolittle Hydropathy [77], Mouse Genome Informatics [64], NCBI [62], PSORT [78], The Connexin-Deafness Homepage [79], SWISSPROT [80], STRING [22], BioGRID [81], and WebGestalt [82].

The Connexin family (pfam00029) comprises 215 members (updating of August, 2016, https://www.ncbi.nlm.nih.gov/Structure/cdd/cddsrv.cgi?uid=pfam00029). RefSeq accession numbers from protein full-length sequences of five human and five mouse paralogues from each CX group A or B were retrieved and used in multiple sequence alignments at Clustal Omega [83]. The last transmembrane domain of each CX was identified at pfam00029. Alignment of the following 42 amino acids as available was manually finalized and basic residues were highlighted.

### 4.9. Yeast Two-Hybrid Assay

The sequence-verified bait or prey constructs were used in self-activation testing by individually transforming the strain NMY51 (MATa his3Δ200 trp1-901 leu2-3,112 ade2 LYS2::(lexAop)4-HIS3 ura3::(lexAop)8-lacZ ade2::(lexAop)8-ADE2 GAL4) using standard procedures. For the yeast two-hybrid interaction test, bait and prey were employed in co-transformation of the yeast strain NMY51. Interaction was verified by testing for His and Ade activation. Lastly, both bait and prey plasmids were used to co-transform yeast Y2HGold. In the case of bait-prey interaction, the reporter genes (*HIS3* and *ADE2*) were activated and yeast was able to grow on SD–Leu^−^ Trp–His^−^ medium and activate the β-galactosidase expression in the X-gal assay (Creative BioLabs, Shirley, NY, USA).

### 4.10. Immunoprecipitation and Western Blotting

Whole livers from P2–P3 mice were lysed in EGTA buffer as described above or in RIPA buffer [50 mM Tris-HCl, pH 7.4, 150 mM NaCl, 50 mM NaF, 5 mM Na_3_VO_4_, 2 mM EGTA, 1% NP-40, 0.1% SDS, 0.5% sodium deoxycholate, 1X protease inhibitor (cOmplete, EDTA-free, Sigma-Aldrich)]. The protein quantity was estimated using a Bradford reagent at 595-nm absorbance. For immunoprecipitation, lysates were precleared in a 1:1 mixture of protein-A and protein-G conjugated to sepharose beads (GE Healthcare) and 1:50 volume of normal mouse serum. Nearly 500 µg of precleared lysates were submitted to incubation with 2 µg of anti-CGN specific antibodies or normal mouse serum for 16 h at 4 °C under rocking. The antibody-lysate mix was then transferred to a microtube containing a 1:1 mixture of protein-A plus protein-G beads (GE Healthcare). Further agitation was at 4 °C for two hours. Beads were pelleted at 8000× *g* for three minutes at 4 °C, washed twice in 50 mM Tris-HCl, pH 7.4, 150 mM NaCl, 50 mM NaF, 5 mM Na_3_VO_4_, and suspended in sample buffer (2% SDS, 100 mM dithiothreitol, 10% glycerol). Western blotting was performed by submitting samples to electrophoresis (6% or 14% SDS-PAGE) and electro-transferring proteins to a 45-µm nitrocellulose filter (BioRad, Hercules, CA, USA) for 16 h at 25 Volts. Transfer efficiency was observed after 1.5% Ponceau S staining. Proteins were blocked for one hour in 1% casein (Novagen) and was followed by 10 min in 3% hydrogen peroxide. Blots were incubated with primary antibody followed by secondary antibody for 1 h each at room temperature. Antibody dilutions were in 2% immunoglobulin-free bovine serum albumin (BSA, Jackson ImmunoResearch Laboratories) in TBS-T (20 mM Tris pH 7.6, 135 mM NaCl, 0.05% Tween-20). All washes were in TBS-T. The filter was incubated in ECL™ Plus (GE Healthcare) and exposed to Amersham Hyperfilm™ ECL film (GE Healthcare).

### 4.11. Indirect Immunofluorescence

Isolated P60 mouse livers were post-fixed in 4% paraformaldehyde for 6 h at 4 °C and then incubated in increasing concentrations of sucrose up to 30% sucrose for 48 h at 4 °C. After inclusion immersion in Jung Tissue Freezing Medium (Leica Biosystems, Buffalo Grove, IL, USA) for two hours, the tissue was frozen and cryo-sectioned in 12-µm histological sections. Isolated cochleae were punctured in the apex for perfusion of 4% paraformaldehyde and was followed by fixation in 4% paraformaldehyde at 4 °C for 16 h. The tissues were then immersed in decalcification solution (10% EDTA and 1% paraformaldehyde) at 4 °C for four days. After washing in PBS, all cochleae were immersed in 10% sucrose solution until the tissue sunk and then all cochleae were incubated in 20% sucrose solution at 4 °C for 20 h. Lastly, the cochleae were immersed in Jung Tissue Freezing Medium (Leica Biosystems) for two hours. The tissue was frozen and cryo-sectioned in 12-µm histological sections (Leica CM-1850, Leica Biosystems).

The histological slides were incubated in acetone at −20 °C for ten minutes and permeabilized in 0.3% triton-X-100 for 20 min, which was followed by 20 min in 0.1 M glycine. Blocking was performed in 5% normal donkey serum in PBS, supplemented with 1× donkey anti-mouse Fab’ (Jackson ImmunoResearch Laboratories). Primary antibody incubation was for 16 h and secondary antibody incubation followed for three hours. All washes were in PBS and antibody dilutions were in 5% normal donkey serum and 0.1% triton-X-100. After the final wash, slides were mounted in DAPI-containing mounting medium (Invitrogen) and sealed after a coverslip was placed. Images were captured in a LSM 880 confocal microscope (Carl Zeiss) after a background fluorescence intensity subtraction from a negative control section (omission of primary antibody) obtained in the same experiment. Pseudo colors and zooming-in were obtained by the ZEN software (Version 2.5, Zeiss Efficient Navigation, Oberkochen, Germany).

## Figures and Tables

**Table 1:** Candidate proteins to interact with CX26 are classified as a cell junction or cytoskeleton-associated proteins.

Human Gene Acronym	Human Gene	Protein Name, Aliases, and Acronyms
*CGN*	Cingulin	Cingulin
*DAAM1*	Disheveled-associated activator of morphogenesis 1	DAAM1
*FLNB*	Filamin B	Filamin-B, Filamin-3, β-filamin
*GAPDH*	Glyceraldehyde-3-phosphate dehydrogenase	GAPDH
*HOMER2*	Homer scaffold protein 2	Homer-2, cupidin
*JUP*	Junction plakoglobin	Plakoglobin, γ-catenin, desmoplakin-3
*MAP7*	Microtubule-associated protein 7	MAP7, EMAP-115, ensconsin
*MAPRE2*	Microtubule-associated RP/EB family member 2	EB2
*PTK2B*	Protein tyrosine kinase 2β	Focal adhesion kinase 2, FAK2, PYK2, PTK2B
*RAI14*	Retinoic acid-induced 14	RAI14, ankycorbin, NORPEG
*TJP1*	Tight Junction Protein 1	*Zonula occludens* 1, ZO-1, TJP1
*VCL*	Vinculin	Vinculin, VCL
